# Highly efficient heritable targeted deletions of gene clusters and non-coding regulatory regions in *Arabidopsis* using CRISPR/Cas9

**DOI:** 10.1038/s41598-018-22667-1

**Published:** 2018-03-13

**Authors:** Julius Durr, Ranjith Papareddy, Keiji Nakajima, Jose Gutierrez-Marcos

**Affiliations:** 1 School of Life Sciences, University of Warwick, Coventry, CV4 7AL UK; 2 Graduate School of Biological Sciences, Nara Institute of Science and Technology, Nara, 630–0192 Japan; 3 PRESTO, Japan Science and Technology Agency, Saitama, 332–0012 Japan

## Abstract

Genome editing using CRISPR/Cas9 is considered the best instrument for genome engineering in plants. This methodology is based on the nuclease activity of Cas9 that is guided to specific genome sequences by single guide RNAs (sgRNAs) thus enabling researchers to engineer simple mutations or large chromosomal deletions. Current methodologies for targeted genome editing in plants using CRISPR/Cas9 are however largely inefficient, mostly due to low Cas9 activity, variable sgRNA efficiency and low heritability of genetic lesions. Here, we describe a newly developed strategy to enhance CRISPR/Cas9 efficiency in *Arabidopsis thaliana* focusing on the design of novel binary vectors (pUbiCAS9-Red and pEciCAS9-Red), the selection of highly efficient sgRNAs, and the use of direct plant regeneration from induced cell cultures. Our work demonstrates that by combining these three independent developments, heritable targeted chromosomal deletions of large gene clusters and intergenic regulatory sequences can be engineered at a high efficiency. Our results demonstrate that this improved CRISPR/Cas9 methodology can provide a fast, efficient and cost-effective tool to engineer targeted heritable chromosomal deletions, which will be instrumental for future high-throughput functional genomics studies in plants.

## Introduction

Phenotypic alterations caused by genetic lesions are the most widely used approach to study gene function in eukaryotes. Over the last century, forward mutagenesis screens have been conducted in several plant species taking advantage of the mutagenic activity of chemicals, ionizing radiation and the mobility of transposable elements^1–3^. However, over the last three decades significant advances in plant transformation technologies have led to the majority of mutagenesis studies exploiting the random integration of foreign transfer DNA (T-DNA)^4,5^. Using the latter approach, extensive mutant libraries have been generated in different plant species^6–8^. However, the major limitations of T-DNA and other traditional mutagenesis strategies are that they are time-consuming to confirm the insertion, collections are largely incomplete due to not all genes in the genome being disrupted, and combining multiple mutations is difficult for those genes that are genetically linked. To overcome some of these limitations, plant researchers have employed reverse genetic screens using transgenes that induce gene silencing by RNA interference (RNAi) or viruses causing viral-induced gene silencing (VIGS)^9^. These methodologies are rapid and efficient in down-regulating gene expression irrespective of gene copy number or chromosomal position, however they normally cause a wide range of hypomorphic phenotypes that can complicate functional studies^10^.

The recent development of targeted gene editing technologies offers significant advantages over the aforementioned methods. Targeted mutations in plants have been generated using zinc-finger-nucleases (ZFN)^11^ and transcription activator-like effector nucleases (TALEN)^12^. Both TALENs and ZFNs encode in their amino acid sequence the specific DNA binding motifs, thus their sequence recognition domains must be designed to target each specific sequence^13,14^. In the last decade, the discovery of a microbial adapted immune system based on the *clustered regularly interspaced short palindromic repeats-Cas* (CRISPR/Cas9)^15–19^ has revolutionised genome editing technology by offering a user-friendly tool for use in microbial, plant and animal species^20–22^. The CRISPR/Cas9 system functions by recognizing genomic target sites through a single chimeric guide RNA (sgRNA)^21^. Chimeric sgRNAs commonly used for CRISPR/Cas9 contain a 17- to 20-nt long variable region, the protospacer, which is complementary to the targeted genomic sequence^23^. The simplicity of designing sgRNAs to recognize different target sequences is one of the biggest advantages of the CRISPR/Cas9 system over previous gene targeting methodologies. The feasibility of CRISPR/Cas9 mediated genome editing has been demonstrated in several plant species, primarily for the generation of targeted loss-of-function mutants through point mutations or small deletions at genes of known function^24–33^. However, the efficiency and heritability of CRISPR/Cas9 genetic lesions in plants is far from optimal with regards to high throughput functional studies^34^.

In the present study, we show how current methodologies for CRISPR-mediated genome targeted deletions in Arabidopsis can be inefficient due to variable sgRNA activity and low heritability. We further demonstrate how the standard CRISPR/Cas9 methodology could be improved to overcome these caveats and aid the rapid and cost-effective generation of targeted heritable chromosomal deletions, thus enhancing further the potential scope for functional genomic analyses in plants.

## Results

### A new methodology for the rapid generation of targeted chromosomal deletions in Arabidopsis

**Figure 1:**
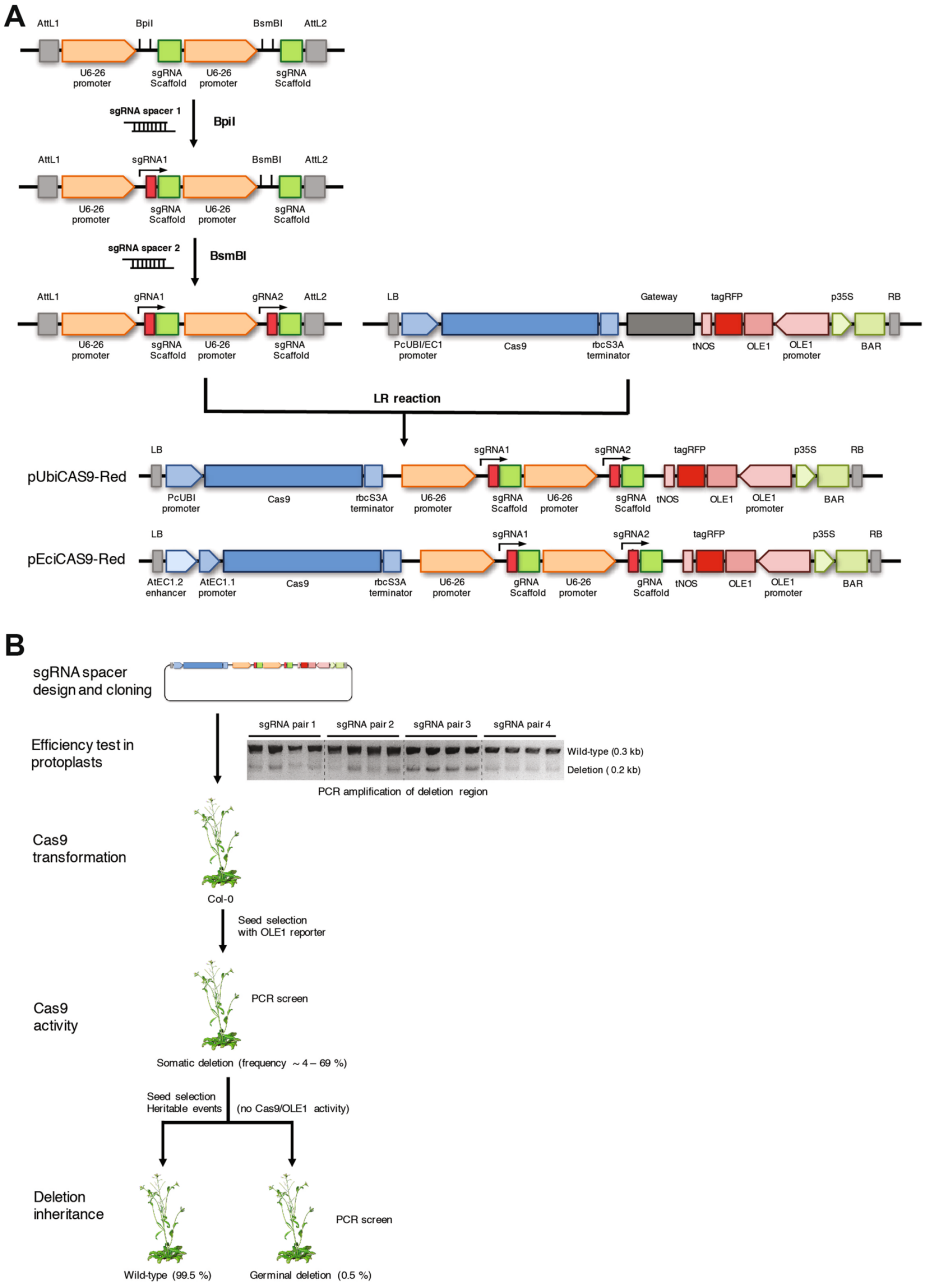
Methodology for the generation of targeted chromosomal deletions in *Arabidopsis*. (**A**) Schematic assembly of the CRISPR/Cas9 vectors. Briefly, 24 nt DNA oligos containing the sequences of the two sgRNAs were used to generate a double-stranded DNA molecule and cloned into the entry vector with the two restriction enzyme sites BpiI and BsmBI, respectively. A LR reaction between the entry vector containing the sgRNAs and the binary vector generated the final Cas9 expression vector. (**B**) The efficiency of the Cas9 expression vector was determined by transfection of *Arabidopsis* protoplast. Selected vectors were integrated using floral dipping. Positive transformants were selected using a seed-specific fluorescent reporter. Cas9 nuclease activity in independent transgenic lines was confirmed by PCR using oligos flanking the deletion. Heritable deletion events were identified in plants lacking Cas9 activity after selection for seeds lacking the seed-specific fluorescent reporter. PCR was carried out using primers JD460 and JD461.

To generate heritable CRISPR/Cas9 chromosomal deletions in plants we created two novel binary vectors named pUbiCAS9-Red and pEciCAS9-Red. For both vectors we used the backbone of pDE-CAS9^29^ (Fig. 1A). Both contain a codon optimized Cas9 driven by the *Petroselinum crispum* Ubiquitin4–2 promoter (PcUbi4–2) or by the synthetic promoter comprising the EC1.2 enhancer fused to the EC1.1 promoter^35^. To aid the rapid insertion of multiple synthetic Cas9 sgRNAs we included an *att*R1-*att*R2 Gateway cassette and generated an entry vector containing two Arabidopsis U6–26 RNA polymerase III promoters flanked by a Cas9 small guide RNA (sgRNA) scaffold flanked by a type IIS restriction enzyme site. This design allows for a rapid and PCR-free direct cloning of two or more chemically synthesized sgRNAs by Golden-Gate assembly^36^ (Fig. 1A). To facilitate positive/negative selection of Cas9-containing plants we included, additionally to the existing bialaphos resistance (BAR) gene in the vector, which confers tolerance to glufosinate^37^, a seed-specific fluorescent reporter^38^ (Fig. 1A). These reporter allows a fast and non-destructive selection of Cas9-negative plants without the need for molecular genotyping. However, it is possible that some lines may carry partial integrations of the transgene.

To assess the efficiency of CRISPR/Cas9 in generating genomic deletions, Arabidopsis plants were transformed with the pUbiCAS9-Red vector containing sgRNA pairs targeting twelve distinct genomic regions. Transgenic plants (T1) were selected based on both bialaphos resistance and the presence of RFP fluorescence in mature seeds (Fig. 1B). To assess the efficiency of the constructs, we grew ~100 independent T1 transgenic lines for each construct and extracted DNA from flower buds to PCR-amplify the targeted genomic region (Supplementary Table 1). This analysis revealed that the targeting efficiency of our Cas9 system in somatic tissues differed dramatically between constructs (4–69%, n = 1,200) (Supplementary Table 1), suggesting that the design of efficient RNA guides is a critical parameter for obtaining high efficiency CRISPR/Cas9 genomic deletions. Because of this, we introduced an additional step to first assess the efficiency of sgRNAs by transfecting binary constructs carrying Cas9 and different combinations of *in silico* designed sgRNAs into Arabidopsis mesophyll protoplasts. We monitored RNA guide efficiency by semi-quantitative PCR amplification of targeted genomic regions using flanking oligonucleotides (Fig. 1B) and determined the extent of the lesions by sequencing. Using this strategy we identified highly efficient sgRNAs, to target discrete chromosomal regions containing large gene clusters, or non-coding intergenic genome regions.

A significant issue with genome editing using CRISPR/Cas9 in plants is the limited heritability of genetic lesions. To circumvent this problem, we selected pUbiCAS9-Red transgenic T1 plants that displayed a high frequency of somatic deletions (Fig. 1B). Seed progenies from these plants were screened for lack of RFP signal in dry seeds to identify those lacking Cas9 activity. Around 100–300 plants for each line were grown in soil and genotyped by PCR to confirm the inheritance of deletions (Fig. 1B). We found that the frequency of these heritable deletions oscillated between 0.5–5% and we were able to create heritable lines only for two out of the twelve regions targeted (Supplementary Table 1). Collectively, our data show that although CRISPR/Cas9 is a suitable tool for generating heritable genomic deletions in Arabidopsis, its efficiency is very low. Recent reports suggest that the efficiency of single mutations created by Cas9 in plants can be improved by the use of promoters that are highly active during early stages of sexual reproduction^35,39^, We therefore modified our vector to drive Cas9 expression using a previously described synthetic EC1 promoter that confers expression in egg cells^35^ and named this vector pEciCAS9-Red. We found that when using this vector the efficiency of heritable deletions increased dramatically (6–100%) for some of the regions targeted. Intriguinly, in some cases we were able to generate offspring carrying homozygous genome deletions, (Supplementary Table 1). These data demonstrate that the use of pEciCAS9-Red vector enhances the efficiency of CRISPR/Cas9 for targeted genome deletions in Arabidopsis.

### Targeted deletions of gene clusters and intergenic regulatory sequences to aid functional analyses in Arabidopsis

**Figure 2:**
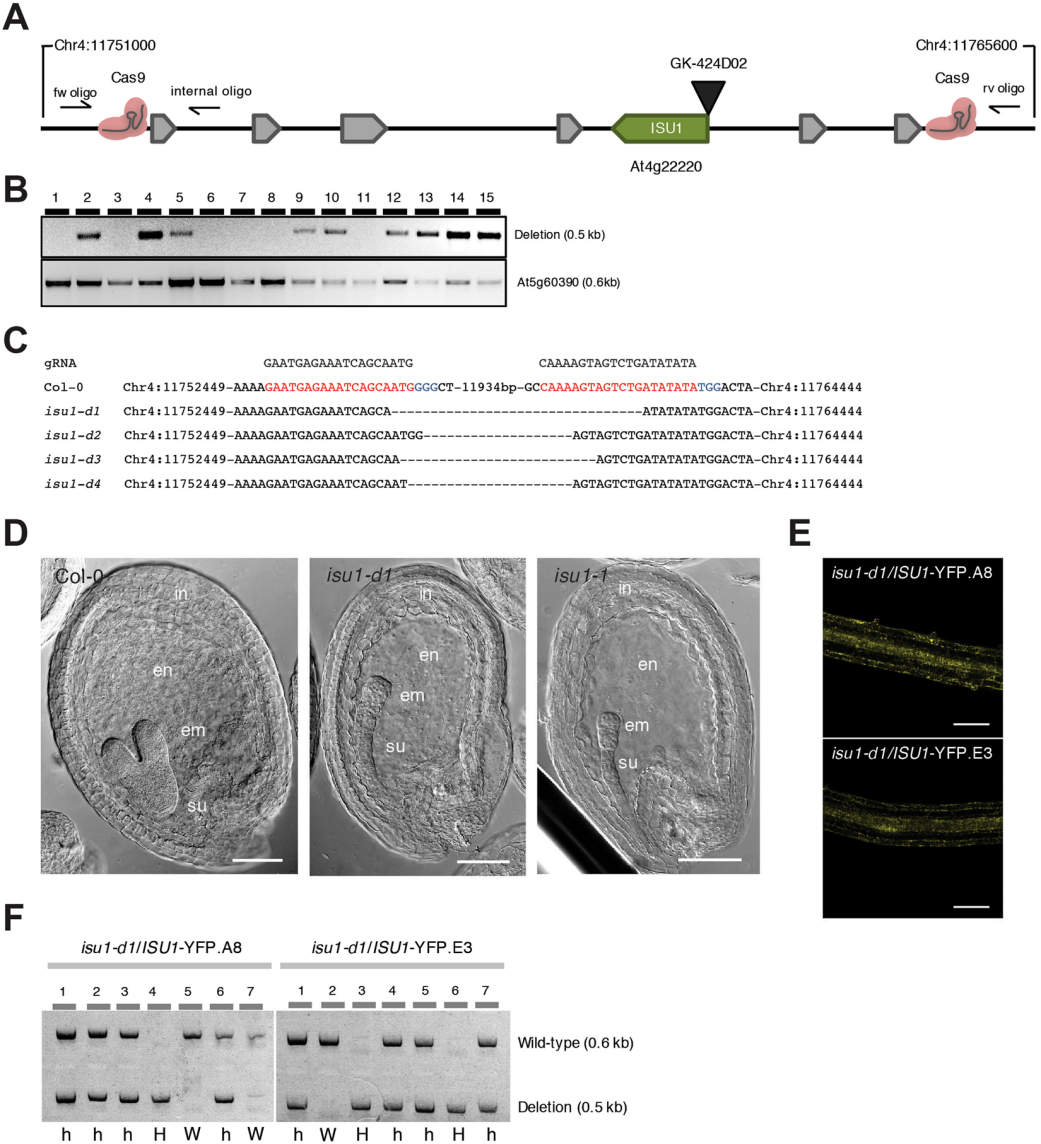
CRISPR/Cas9 targeted deletions of the *ISU1* locus and genetic complementation with an ISU1-YFP reporter. (**A**) Schematic representation of the *ISU1* locus and the Cas9 binding sites. (**B**) PCR analysis of the *ISU1* locus with oligonucleotides flanking the deletion site. The expected PCR size containing a deletion is 500 bp. Primers JD157 with JD158 and JD125 with JD126 were used for the *ISU1* deletion and At5g60390 control, respectively. (**C**) Sanger sequencing results of four independent deletions (T1) (sgRNA sequence in red and PAM sequence in blue). (**D**) Defects in early embryo development associated with *isu1-d1* and *isu1–1* (GK424D02) mutations. Scale bar: 50 μm. in, integuments, en, endosperm, em, embryo, su, suspensor (**E**) Confocal microscopy images of roots from two independent ISU1-YFP transgenic lines that complement the *isu1-d1* mutant. Scale bar: 50 μm. (**F**) Genotype analysis of plants from two independent *isu1-d1*/ISU1-YFP complemented lines. Deletions of the *ISU1* locus was determined by PCR using oligonucleotides flanking the deletion site. The expected PCR product size for the deletion is 500 bp and the expected PCR size for the wild-type ISU1 allele is 600 bp. Primers JD157 with JD159 and JD157 with JD158 were used for the ISU1 wild-type allele and ISU1 deletion allele, respectively. W, wild-type; H, homozygous; h, heterozygous.

To demonstrate the efficacy of CRISPR-mediated genomic deletions for functional genomic studies, we first selected a genomic region flanking the *ISU1* locus, which also contains a large cluster of defensin-like genes (Fig. 2A). *ISU1* encodes a scaffold protein targeted to mitochondria that is involved in iron-sulfur cluster biogenesis^40,41^. It has been previously shown that RNAi downregulation and T-DNA promoter insertions in *ISU1* cause a wide range of developmental phenotypes^42^. We decided to target the full *ISU1* locus by removing a ~12 kb segment that includes coding and regulatory sequences (Fig. 2A). We designed different sgRNA pairs that were tested in *Arabidopsis* protoplast transfections and selected pairs based on their targeting efficiency to generate two ISU1-Cas9 binary constructs using our pUbiCAS9-Red binary vector. We grew 152 independent transgenic lines, genotyped these lines with oligos flanking the expected deletion region and found that 77 plants displayed targeted deletions (51%) (Fig. 2B and Supplementary Table 1), which we further confirmed by sequencing (Fig. 2C). We then selected 396 Cas9-negative seed progenies from nine independent lines and identified two independent heritable events, which we named *isu1-d*. When comparing T-DNA exonic insertional mutant plants (*isu1–1)* and Cas9 deletion plants (*isu1-d*) we found that we could not recover homozygous plants from either population. We then analyzed mature siliques and found that a proportion of the seeds (23.5%, n = 336) displayed a significant reduction in size and in some cases abnormal morphology. To assess the nature of these defects we analyzed developing seeds in heterozygous insertional and targeted deletion mutants, and found that for both mutant lines early globular embryo development was arrested in approximately a quarter of the seeds (24.6%, n = 455) indicating that these mutations are recessive and cause post-zygotic defects during seed development. In particular, globular embryos possessed disorganized apical embryonic cells while extra-embryonic suspensor cells developed normally (Fig. 2D). To confirm that these phenotypes where directly caused by the loss of ISU1 function, we complemented *isu1-*d plants with a translational ISU1-GFP reporter. We found that out of 22 independent lines tested, six fully restored embryo development and seed viability, which then allowed us to recover homozygous *isu1*-d/ISU1-GFP plants (Fig. 2E and F).

**Figure 3:**
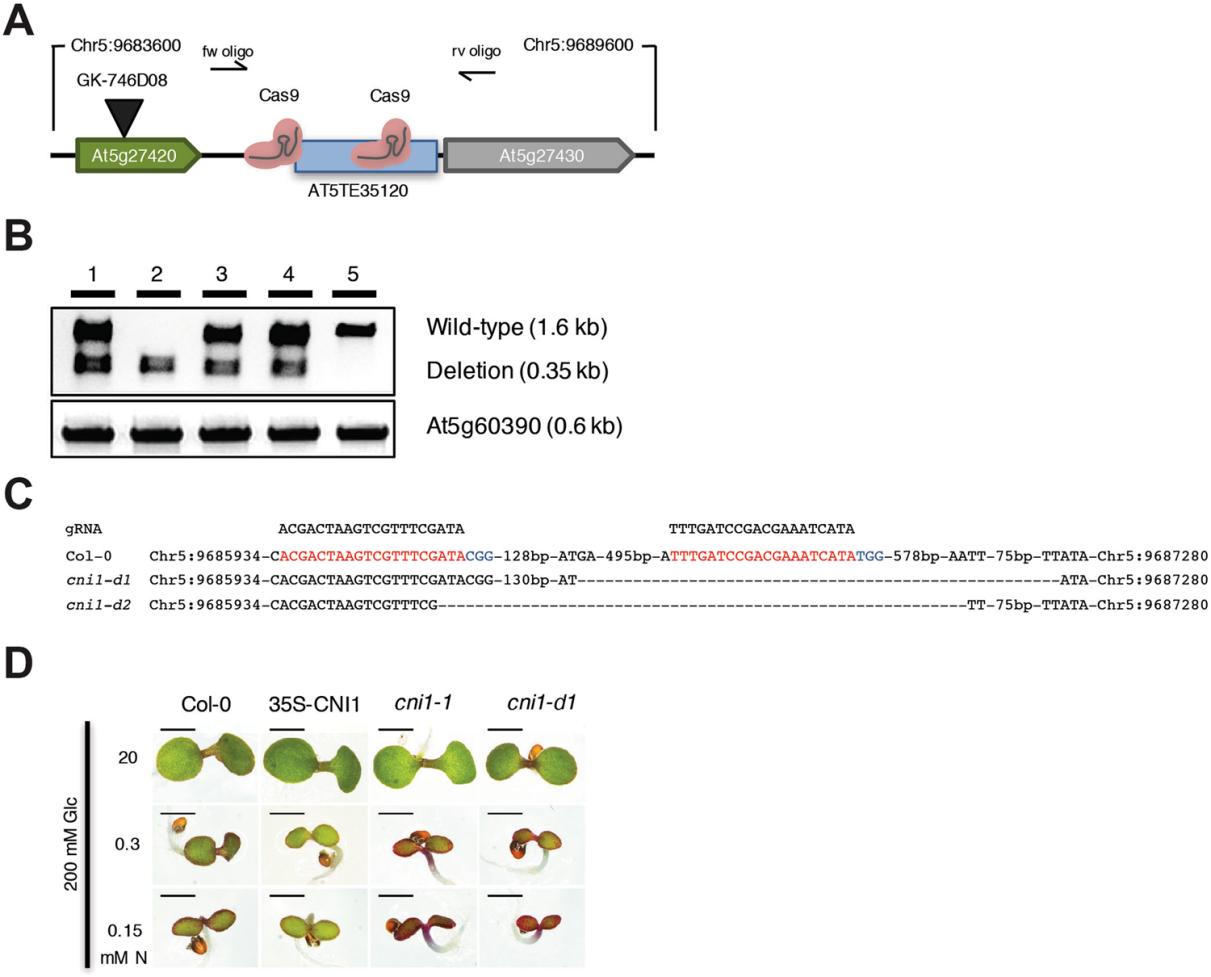
CRISPR/Cas9 targeted deletion of the *CNI1* intergenic regulatory region. (**A**) Schematic representation of the *CNI1* locus and sequences targeted by Cas9. (**B**) PCR analysis of plants segregating for heritable deletion line *cni1-d1* (T2) (*cni1-d*) with oligonucleotides flanking the deletion site. The expected PCR size of the deletion allele is 350 bp, the expected PCR size for the wild-type allele is 1.6 kb. Primers JD271 with JD272 and JD125 with JD126 were used for the *CNI1* deletion and At5g60390 control, respectively. (**C**) Sanger sequencing results of two independent heritable *cni1-d* lines (T2) (sgRNA sequence in red and PAM sequence in blue). (**D**) Post-germinative growth phenotype of *cni1-d1* compared to 35S-CNI1, *cni1–1* mutants and WT after growth (4 days after germination) on medium containing different concentrations of nitrogen (0.15–20 mM) with 200 mM glucose. Scale bar: 1 mm.

We next tested the application of our CRISPR/Cas9 genome targeting approach to dissect the function of a non-coding regulatory region. For this analysis we selected the intergenic non-coding regulatory region of *CARBON/NITROGEN INSENSITIVE 1* (*CNI1*)- a plant specific ubiquitin ligase critical for carbon and nitrogen homeostasis, defense response and leaf senescence^43–45^. The region targeted comprises a *cis* regulatory element located in a transposable-related element, which is sensitive to epigenetic modification in response to stress^46^. We selected sgRNA pairs by *Arabidopsis* protoplast transfection and generated two distinct pUbiCAS9-Red binary constructs to target this genomic region (Fig. 3A). We found that both constructs were highly efficient in generating targeted deletions in somatic tissues (Supplementary Table 1) and allow us to identify two independent heritable deletions that we named *cni1-d* (Fig. 3B). These two deletion lines showed the expected genetic segregation and sequence analysis confirmed that these lines carried independent genomic lesions (Fig. 3C). Interestingly, both heritable lines show different larger additonal deletions adding to a deletion size of ~1.2 kB compared with the expected 0.66 kb. Because CNI1 participates in carbon and nitrogen sensing, we grew homozygous *cni1-d1* plants in media containing varying concentrations of nitrogen and glucose. We also included in our analysis a homozygous T-DNA insertion mutant line (*cni1–*1) and a transgenic line that constitutively express *CNI1* (35S-CNI)^44^. We found that under limiting nitrogen and high glucose growth conditions, both *cni1-d1* and *cni1–1 plants* accumulated strong purple pigmentation and displayed arrested growth phenotypes. On the other hand, wild-type and 35S-CNI1 plants did not show strong accumulation of pigments and/or growth arrest. Collectively, our experiments demonstrate that highly efficient CRISPR/Cas9 targeted genome-editing methodology can enhance significantly functional genomic studies in plants.

### A new strategy to increase the heritability rates of targeted CRISPR/Cas9 genomic deletions in Arabidopsis

**Figure 4:**
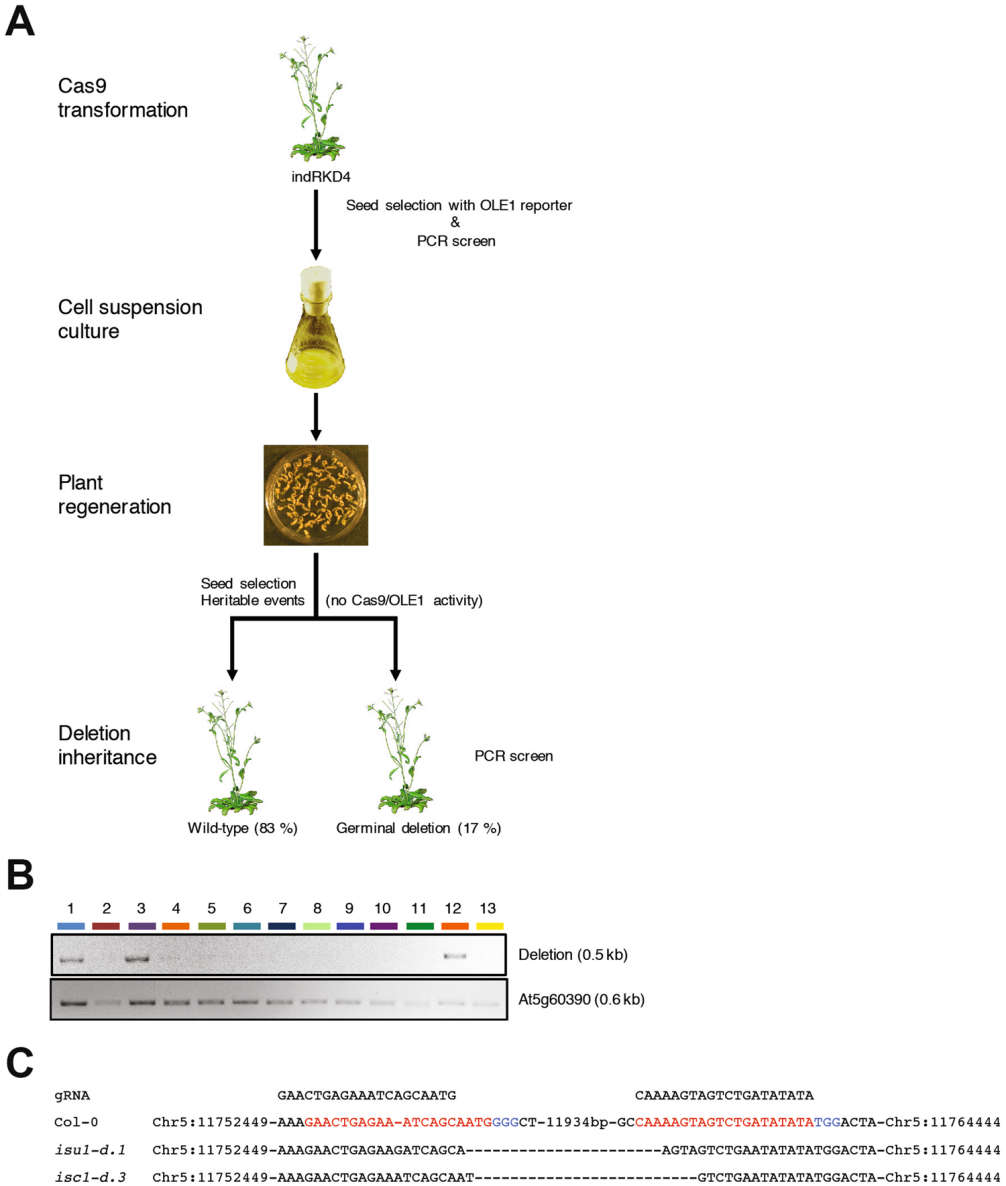
A novel strategy for highly efficient CRISPR/Cas9 targeted genomic deletions in Arabidopsis. (**A**) The Cas9 expression vector was integrated into indRKD4 plants using floral dipping. Positive transformants were selected using the fluorescent seed-specific reporter. Positive seeds were grown in liquid medium containing dexamethasome. After two weeks cell suspensions were transferred to plates containing standard medium. Regenerated plants were tested for Cas9 nuclease activity by PCR and selection of heritable deletion was determined in plants lacking Cas9 activity. Primers JD157 with JD158 and JD125 with JD126 were used for the *ISU1* deletion and At5g60390 control, respectively. (**B**) PCR analysis of the *ISU1* locus to identify targeted deletions. (**C**) Sanger sequencing results of plants containing independent *ISU1* heritable deletions.

Our work has revealed that it is possible to generate heritable targeted genomic deletions in Arabidopsis using CRISPR/Cas9, although its efficiency is limited by the efficacy of the selected sgRNA, optimal Cas9 activity, and multigenerational analysis of modified plants (Fig. 1). Another major limiting factor of this approach is the reduced transmission of genetic lesions to the offspring. Because chromosomal deletions require the dual targeting of Cas9 to two distant sequences on the same chromatin, this could be particularly problematic in meristematic cells, which are known to have active DNA repair mechanisms that could affect targeting efficiency^47^. To test this hypothesis, we generated CRISPR/Cas9 lines to induce *ISU1* genomic deletions in somatic cells and then recovered these lesions by induced plant regeneration. To facilitate the rapid and cost-effective regeneration of plants from somatic cells we generated a transgenic line expressing the zygotic transcription factor RKD4^48,49^ under the regulation of a chemically inducible system, which we named indRKD4. We transformed indRKD4 plants with a pUbiCas9-Red construct designed to target the *ISU1* locus with high efficiency in somatic cells (Fig. 4A). We identified forty primary transformants and selected five for analysis based on their ability to generate *ISU1* somatic deletions. From each of the lines we selected 100 Cas9 positive seeds that were placed in liquid media (containing dexamethasone as a chemical inducer) to generate somatic cell suspension cultures. Two weeks after culture, cell suspensions where transferred to solid medium (devoid of dexamethasone) to induce plant regeneration (Fig. 4A). We selected five independent regenerants per line from where we collected ten Cas9-negative dry seeds (10/line). We grew plants from these seeds to identify those carrying heritable ISU1 deletions (Fig. 4B). Almost one fifth of these plants (17%, n = 250) carried independent *ISU1* chromosomal deletions, which we confirmed by sequencing (Fig. 4C). Thus through this approach of combining our pUbiCAS9-Red vector with direct plant regeneration from indRKD4 somatic cell cultures we could improve the efficiency of targeted genomic deletions and generate a large number of independent heritable genetic lesions.

## Discussion

### A highly efficient method for targeted chromosome deletions using CRISPR/Cas9 in plants

Newly developed targeted genomics tools, such as ZFN, TALEN and Cas9, have opened the possibility of rapidly generating precise, customized genetic lesions in plants. These methodologies can be applied to genes of unknown function but also to genes for which the function is not fully understood because mutants generated using classic mutagenesis may contain unwanted genetic lesions or chromosomal rearrangements that could interfere with their functional analyses^50,51^. For instance, CRISPR-generated loss-of-function alleles of the *AUXIN BINDING PROTEIN 1* (*ABP1*) in Arabidopsis have revealed that phenotypes initially described in T-DNA alleles were inaccurate^52^. The CRISPR/Cas9 system has also a great potential for promoting functional studies in plants, as it can be easily adapted to generate true loss-of-function mutations for single genes, large gene clusters and also non-coding regulatory sequences. In mammals, single Cas9–sgRNA tiling approaches have been developed to dissect the activity of regulatory elements demonstrating the function of enhancer sequences and their role in gene expression regulation^53–55^. However, the use of tiling Cas9-sgRNA in plant functional analysis is not straightforward. We instead opted for an alternative approach based on the capacity to create stable targeted deletions of regulatory elements using CRISPR/Cas9. Our work demonstrates for the first time that this approach is suitable for the functional characterization of distantly acting regulatory sequences in plants. This methodology could be further adapted to engineer targeted epigenetic alterations, such as histone modifications or DNA methylation, using catalytically inactive Cas9 (dCas9)^56–60^.

A major limitation for the use of CRISPR/Cas9 in functional genomic studies in plants is the difficulty to generate meiotically stable genetic lesions with high efficiency. Our work has revealed that the generation of chromosomal deletions using current methodologies is highly inefficient. The low efficiency of this technique in Arabidopsis could be attributed to several factors; an important factor being the unpredictable efficiency of the *in silico* designed sgRNAs. Although there are several computational resources that aid the design of highly specific sgRNAs to minimize off-target effects in plants^61–63^ their efficiency *in vivo* cannot be predicted. Recent studies in animals have shown that the efficiency of the sgRNA depends on the nucleotide composition of the protospacer sequence^64–67^. In contrast, a recent study in rice was unable to detect nucleotide preferences for a collection of sgRNA designed for Cas9 gene targeting^68^. Although protospacer sequences with high GC-content have been associated with higher Cas9 editing efficiencies^69^, no direct link to nucleotide preference could be drawn in plants. By first testing the efficiency of sRNAs *in vivo* using protoplast transfection, our strategy offers a fast, simple and high-throughput method for CRISPR/Cas9 functional genomic analysis in plants. A second parameter that influences the efficiency of heritable genomic deletions in Arabidopsis is the promoter used to drive Cas9 expression. We found that a promoter that drives high expression in somatic cells is highly efficient in generating genomic deletions, however these lesions often exhibit low heritability. Our data show that the presence of Cas9-derived genomic deletions in somatic tissues is not a good proxy for strong germline transmission. This may be attributable to differences in DNA repair for somatic and meristematic cells, because heritable Cas9 genome deletions require simultaneous editing activity of the two sgRNAs. These results may be linked to the reported mechanisms that plants have developed to safeguard the genetic integrity of meristematic cells^47^. By generating Arabidopsis lines expressing the zygotic factor RKD4, we were able to combine Cas9-metiated genome editing in somatic cells and direct plant regeneration, and found that the efficiency of Cas9-mediated genome deletions increased. We foresee that this methodology could make possible CRISPR/Cas9 high-throughput functional studies in crop plants where plant regeneration from cell cultures has been already demonstrated^70–74^. Our methodology could also be potentially enhanced using transfection or viral infection as a transgene-free approach for the delivery of sgRNA/Cas9 in plants^75–77^. Several studies have shown that CRISPR/Cas9 editing efficiency can be improved when using promoters active during different stages of plant sexual reproduction^35,78–80^. We therefore adapted our system to drive Cas9 expression using a synthetic promoter that is preferentially expressed, although not exclusively, in Arabidopsis egg cells. We found that combining the use of a synthetic egg cell promoter with the *in vivo* sgRNAs screening enhances the efficiency of Cas9 targeted genome deletions. Moreover, we found that some plants produced progenies that were homozygous for the targeted deletion, which indicates that the promoter is active throughout embryo development thus minimizing the formation of chimeric mutations. Since both of the promoters tested - the U6–26 and the synthetic EC1 promoter - are active in Arabidopsis and other dicotyledonous plants^27,81–83^, we anticipate that our genome editing strategy can be directly applied to other plant species.

One widely reported disadvantage of CRISPR/Cas9 genome editing for functional genomic studies is the occurrence of off-target mutations^22,84,85^, with the frequency of off-targets in mammals being higher than plants^86–88^. Various effort has been made to overcome such limitations, ranging from the identification of new CRISPR/Cas systems^89,90^ to the development of newly engineered Cas9s^91,92^ Although we have not determined the frequency of off-targets in our CRISPR/Cas9 genome deletions, the generation of independent genetic lesions using different sgRNA pairs coupled to multigenerational propagation by selfing or backcrossing should minimize the impact of off-targets.

In summary, we have established an efficient and cost-effective platform for the generation of heritable targeted chromosomal deletions in Arabidopsis using CRISPR/Cas9. We demonstrate that the high efficiency of our system is based on (i) an improved methodology for initially selecting highly efficient sgRNAs, (ii) expression of Cas9 during early stages of sexual reproduction and (iii) propagating deletions occurring in somatic cells by direct plant regeneration. The optimization of these methodologies will be fundamental to facilitate high-throughput functional studies in crops and plants with complex genomes.

## Material and Methods

### Plant lines and growth conditions

All plant material used in this study was derived from the wild-type Columbia (Col-0) accession. The *isu1–1* mutant (GK_424D02) was obtained from GABI-Kat collection^93^. The *cni1–1* mutant and CNI1 overexpression lines have been reported previously^46^. Mutant and wild type (Col-0) seeds were sown on soil (John Innes and Perlite mix), stratified for 2 days at 4 °C in the dark, and grown at 20 °C under long photoperiod conditions (150 µmol/m2/s and 16 h/8 h light/dark cycles) to induce flowering. Seeds were surface sterilized for 3 min in 70% ethanol, followed by a 2 min treatment in 1% NaOCl. Seeds were then washed six times in sterile H_2_O, dispersed in sterile 0.1% agarose and placed on half-strength MS medium (Murashige & Skoog, Duchefa), solidified with 0.8% Phytagar.

### Plant transformation and protoplast

Arabidopsis plants were transformed via Agrobacterium-mediated transformation as described previously^5^. Arabidopsis mesophyll protoplasts for transient expression of the CRISPR/Cas9 construct were prepared as described previously^94^ with minor modifications. Aproximately 80,000 protoplasts were transformed with 16 μg of plasmid (pUbiCAS9-Red) and incubated for 48 h at 22 °C under long photoperiod conditions (150 µmol/m2/s and 16 h/8 h light/dark cycles). DNA was isolated and concentrations adjusted before performing a semi-quantitative PCR using oligonucleotides flanking the region targeted for deletion (Fig. 1B and Supplementary Table 1).

### T-DNA constructs

The binary vectors used in this study were derived from pDE-CAS9^29,35^. The OLE1-RFP reporter was amplified by PCR using the pFAST-R03 vector^38^ as a template and oligos JD08 and JD09 (Supplementary Table 2). The OLE1-RFP reporter cassette was then cloned in pDE-CAS9 using SpeI and PacI restriction sites, to generate the vector pUbiCAS9-Red. We replaced the UbiCAS9 cassette with the EciCAS9 fragment from pHEE401E (Wang *et al*.^35^) to generate the vector pEciCAS9-Red. We generated a customizable entry vector by modifying the pEN-Chimera vector^29^. The first guide RNA was amplified by PCR using JD01 and JD02 and cloned with SacII and PstI restriction enzymes. A second guide RNA was synthesized by Integrated DNA Technologies, amplified with JD03 and JD04 (Supplementary Table 2). PstI and SacI were used to create the pEN-2xChimera vector. The two protospacer sequences that act as guides to specific target sequence were designed using CRISPR-PLANT^62^, chemically synthetized and cloned into pEN-2xChimera using BpiI and BsmBI restriction enzymes, respectively. The customized guide RNAs were then transferred into pUbiCAS9-Red or pEciCAS9-Red by Gateway® single-site LR recombination-mediated cloning.

The *ISU1* gene including the promoter region was amplified from *A*. *thaliana* genomic DNA using the oligos JD335 and JD336. The PCR fragment was cloned into pDONR207 using Gateway BP clonase (Thermo Fisher Scientific). The insert of the entry clone was them integrated into pGWB440^95^ by LR clonase (Thermo Fisher Scientific).

### Selection of germinal deletions

Primary transformants were selected with the seed specific RFP reporter using a Leica MZ-FL III stereomicroscope (Leica Camera AG). Plants were grown on soil and genomic DNA was extracted form approximately 4 week old plants. For genotyping oligos JD157/JDF158 (deletion) and JD157/JD160 (WT) were used. Progeny were checked for a 3:1 segregation. RFP negative seeds of single locus lines were sown on soil and genotyped for deletion events. Deletions were confirmed using Sanger sequencing.

### Generation of RKD4 lines and direct plant regeneration from cell cultures

A DNA fragment spanning the entire protein-coding and intronic seqences of *RKD4* (*gRKD4*) was amplified from the genomic DNA prepared from *A*. *thaliana* accession Col-0 plants with the primers Apa-RKD4–5′ (5′-ttggggccccagagactatatATGAGTTCGTC-3′) and Cla-Spe-RKD4-CtR (5′-aaatcgatactagTCAATAATAATCATCACC -3′). This fragment was inserted into the pBI-Bar-UAS-NosT-35S-ADH5′-GVG plasmid that contained DNA fragments of pNos-Bar^96^ 5xUAS-NosT (Waki *et al*. ^48^), CaMV35S promoter, tobacco ADH 5′ leader^97^ and Gal4-VP16-GR (GVG)^98^ in the pBIN19 backbone^99^ The resulting plasmid (pBI-Bar-UAS-gRKD4–35S-GVG) was introduced into wild-type plants by the floral dip method, and transgenic plants (named indRKD4) were selected with 8 mg/L of bialaphos. Homozygous indRKD4 plants were transformed by floral dip with pUbiCAS9-Red_ISU1 vector and seeds carrying this transgene were isolated by fluorescent seed selection. Seeds were surface sterilized and germinated in inducing medium containing Gamborg’s B-5 Basal Salt Mixture (Sigma-Aldrich), 2% sucrose, 0.05% MES/KOH (pH 5.7), 10 µg/ml thiamine, 1 µg/ml pyridoxine, 1 µg/ml nicotinic acid and containing 30 µM dexamethasone. After two weeks in culture the cell suspension was spread on solid MS medium (without dexamethasone) to induce the formation of somatic embryos and allowing direct plant regeneration. Regenerated plants were transferred to soil and genotyped by PCR to identify those carrying targeted chromosomal deletions.

### Low nitrogen/High sugar growth assay

Plants were grown on medium containing 0.15 or 0.3 mM KNO_3_, 1.5 mM CaCl_2_, 0.75 mM MgSO_4_, 0.625 mM KH_2_PO_4_ and 200 mM glucose, supplemented with Murashige and Skoog basal salt micronutrient solution (Sigma-Aldrich). ½ MS with 200 mM glucose was used as mock control. Images were taken 5 days after germination. Images were taken using a Zeiss Discovery.V12 stereomicroscope equipped with a Zeiss AxioCam HRc.

### Microscopy analysis of developing seeds

Developing ovules, 3 days after pollination (DAP), were mounted in Hoyer’s solution (3.75 g gun arabic, 25 g chloral hydrate, 1.25 ml glycerol and 15 ml H_2_O). Differential interference contrast (DIC) images were obtained with a Zeiss Axiovert 200 M inverted microscope equipped with a confocal laser-scanning module (Zeiss LSM 710). Image processing was done using Fiji (fiji.sc).

### Data availability

The nucleotide sequences reported in this paper have been submitted to NCBI under GenBank accession numbers KY489664 (pEN-2xChimera), KY489665 (pUbiCas9-Red) and KY489666 (pEciCas9-Red).

## Electronic supplementary material

Supplementary information
