# Supplementary material for: Highly efficient heritable targeted deletions of gene clusters and non-coding regulatory regions in Arabidopsis using CRISPR/Cas9
Source: Sci Rep. 2018 Mar 13;8:4443. doi: 10.1038/s41598-018-22667-1 (PMC5849686; doi:10.1038/s41598-018-22667-1)
Supplement: Supplementary file 1 — Supplementary information [file 41598_2018_22667_MOESM1_ESM.pdf]

Running Title:

Highly efficient heritable targeted deletions of gene clusters and non-coding regulatory regions in *Arabidopsis* using CRISPR/Cas9

Authors and Author's Addresses:

Julius Durr[1], Ranjith Papareddy[1], Keiji Nakajima [2,3] and Jose Gutierrez-Marcos[1*]

[1]School of Life Sciences, University of Warwick, Coventry CV4 7AL, UK
[2] Graduate School of Biological Sciences, Nara Institute of Science and Technology, Nara 630-0192, Japan
[3] PRESTO, Japan Science and Technology Agency, Saitama 332-0012, Japan

*__Correspondence:__

j.f.gutierrez-marcos@warwick.ac.uk
University of Warwick, School of Life Sciences, Coventry CV4 7AL, UK
Tel.: +44 2476 575077

Table 1

sgRNA pairs	sgRNA protospacer 1	SeqID 1	sgRNA protospacer 2	SeqID 2	Size (kb)	T1 total number of lines analysed		T1 number of mutated lines	T1 efficiency	T2 number of lines analysed	T2 total number of plants analysed	T2 number of lines with mutated plants	T2 number of mutated plants	T2 efficiency
CRP0730-1	TAAGAACAGCGAAATTTATG	Chr1:5083775-5083795:c	TAAAAGATAATCTGTACCTG	Chr1:5083822-5083842:c	0.05	PcUbi	80	34	**42%**	-	-	-	-	**-**
						AtEC1	-	-	**-**	-	-	-	-	**-**
CRP0730-2	GAACCATTGGCCAGAAAACG	Chr1:5083959-5083979:c	CAAATGTGTCGCGATTATTA	Chr1:5084002-5084022	0.04	PcUbi	-	-	**-**	-	-	-	-	**-**
						AtEC1	-	-	**-**	-	-	-	-	**-**
CRP0860-1	ACAAGTTTTAATATCATGTA	Chr1:22455180-22455200	ACTACATAAGGACAACACAC	Chr1:22462721-22462741:c	7.54	PcUbi	76	3	**4%**	2	124	0	0	**0%**
						AtEC1	96	4	**4%**	2	80	0	0	**0%**
CRP0860-2	TGTTTAAGTAAGCTATATAT	Chr1:22455118-22455138:c	TTTCTGCATTTATTTTGATT	Chr1:22462680-22462700:c	7.56	PcUbi	-	-	**-**	-	-	-	-	**-**
						AtEC1	96	3	**3%**	2	80	0	0	**0%**
CRP0860-3	TCTCCCAAGCCTAACCATTG	Chr1:22455366-22455386	GGAGTGCAATCACAGTAGAT	Chr1:22462521-22462541:c	7.14	PcUbi	-	-	**-**	-	-	-	-	**-**
						AtEC1	80	10	**12.5%**	3	120	1	8	**6%**
ISU1-1	GAACTGAGAAATCAGCAATG	Chr4:11752450-11752470	CAAAAGTAGTCTGATATATA	Chr4:11764417-11764437	11.97	PcUbi	152	77	**51%**	9	390	2	2	**0.5%**
						AtEC1	-	-	**-**	-	-	-	-	**-**
ISU1-2	TGCAATTTTATATTGGAACT	Chr4:11752249-11752269:c	TTCGGGAGATACACTTTGCA	Chr4:11764850-11764870	12.60	PcUbi	118	38	**32%**	2	160	0	0	**0%**
						AtEC1	-	-	**-**	-	-	-	-	**-**
CRP0680.1-1	TTTATCCTACATGCTGGTGT	Chr3:1368262-1368282:c	AAGAGTTCTTCATTCTTTGT	Chr3:1369519-1369539	1.26	PcUbi	88	40	**45%**	-	-	-	-	**-**
						AtEC1	-	-	**-**	-	-	-	-	**-**
CRP0680.1-2	GCCTGAAGCCTCAAAACATG	Chr3:1367951-1367971	GGATAAGTAGATTCTCTATT	Chr3:1371394-1371414	3.44	PcUbi	82	19	**23%**	3	240	0	0	**0%**
						AtEC1	56	6	**11%**	2	80	2	55	**69%**
CRP0680.2-1	AACGGTTGGTTAGAACCACA	Chr3:8264411-8264431:c	GAATTGATGGTAACCAATTT	Chr3:8267378-8267398	2.97	PcUbi	85	39	**46%**	-	-	-	-	**-**
						AtEC1	80	26	**33%**	-	-	-	-	**-**
CRP0680.2-2	GGATTAGAACTTATTCCATG	Chr3:8264393-8264413	ATGCCACAATCTATGACCAA	Chr3:8267577-8267597:c	3.18	PcUbi	78	21	**28%**	3	120	0	0	**0%**
						AtEC1	40	14	**35%**	3	120	0	0	**0%**
CRP0710-1	CCCCGATCCTTACGGTTCGT	Chr2:9711949-9711969:c	AATACTGGTGCTTATAGAGG	Chr2:9715896-9715916	3.95	PcUbi	60	12	**20%**	-	-	-	-	**-**
						AtEC1	-	-	**-**	-	-	-	-	**-**
CRP0710-2	GATGACGGTTATTGATTGTT	Chr2:9712005-9712025:c	TACAAAGGGTGGAATTCCCC	Chr2:9716153-9716173	4.15	PcUbi	-	-	**-**	-	-	-	-	**-**
						AtEC1	96	11	**11%**	2	80	2	13	**16%**
CRP1120-1	GTTACTAGGTGAATCAAAGA	Chr5:19018024-19018044:c	ATGTCGTTGATGTTCTGTAA	Chr5:19026796-19026816	8.77	PcUbi	40	6	**15%**	-	-	-	-	**-**
						AtEC1	-	-	**-**	-	-	-	-	**-**
CRP1120-2	ATAATAAATTATAGATGTGG	Chr5:19017879-19017899	TAAGTACAAAATGGGAAAAA	Chr5:19026411-19026431	8.53	PcUbi	40	6	**15%**	-	-	-	-	**-**
						AtEC1	-	-	**-**	-	-	-	-	**-**
CRP0580-1	ATTAGGAGTTCTACTAGCTT	Chr4:14425464-14425484	TATAACCTAACGATGGCAAT	Chr4:14437050-14437070	11.59	PcUbi	112	21	**19%**	3	240	0	0	**0%**
						AtEC1	40	1	**3%**	1	80	1	80	**100%**
CRP0580-2	AGCTGCTATACGGGGTACAA	Chr4:14425510-14425530:c	TAGTCAAAAGCCTACTCTTT	Chr4:14436887-14436907:c	11.38	PcUbi	80	3	**4%**	2	160	0	0	**0%**
						AtEC1	40	0	**0%**	-	-	-	-	**-**
CRP1040-1	AGCGGATGTAAATGATTTAT	Chr1:4660711-4660731:c	TGGTTTCTGCTGTGAAACCA	Chr1:4663109-4663129:c	2.40	PcUbi	74	5	**7%**	-	-	-	-	**-**
						AtEC1	-	-	**-**	-	-	-	-	**-**
CRP1040-2	TGAAATTAATACTAAAACAG	Chr1:4659855-4659875:c	GAGTTAAACGGTTGGATTGT	Chr1:4663129-4663149:c	3.27	PcUbi	-	-	**-**	-	-	-	-	**-**
						AtEC1	40	0	**0%**	-	-	-	-	**-**
ESF1-1	CTCTCGGCTAATTGCTTTTA	Chr1:3561652-3561672:c	TTATATCTGCAGGTTTATAA	Chr1:3574908-3574928:c	13.28	PcUbi	74	12	**12%**	-	-	-	-	**-**
						AtEC1	-	-	**-**	-	-	-	-	**-**
ESF1-2	AGATTTGAGGGGAAATCTAT	Chr1:3561487-3561507	ATATACGAATTAGTTAAGCC	Chr1:3574726-3574746	13.26	PcUbi	78	13	**17%**	-	-	-	-	**-**
						AtEC1	-	-	**-**	-	-	-	-	**-**
CNI1-1	ACGACTAAGTCGTTTCGATA	Chr5:9685936-9685956	TTTGATCCGACGAAATCATA	Chr5:9686591-9686611	0.66	PcUbi	79	70	**79%**	2	80	2	4	**5%**
						AtEC1	-	-	**-**	-	-	-	-	**-**
CNI1-2	TGTTGTCTACAATATGATGA	Chr5:9686073-9686093	GATTAATAACTTTGCCATTA	Chr5:9687166-9687186	1.11	PcUbi	58	16	**27%**	-	-	-	-	**-**
						AtEC1	-	-	**-**	-	-	-	-	**-**
MYB20-1	ACCAAACTCTGGTAAGCCGG	Chr1:24675252-24675272:c	TTGAATTTGTTCGGTTGTGA	Chr1:24676551-24676571	1.32	PcUbi	40	6	**15%**	-	-	-	-	-
						AtEC1	-	-	-	-	-	-	-	-
MYB20-2	ATTCTTGTACCGCTCACAAT	Chr1:24675202-24675222:c	ATTGTTACTGGTGCGTGGGA	Chr1:24676574-24676594	1.39	PcUbi	40	5	**13%**	-	-	-	-	-
						AtEC1	-	-	-	-	-	-	-	-

JD01	sgRNA1 fw	GCGGCCGCGGCAAATAATGATTTTATTTTG
JD02	sgRNA1 rv	AATTCTGCAGTAATGCCAACTTTGTACAAG
JD03	sgRNA2 fw	AATTCTGCAGCTTTTTTTCTTCTTCTTCGT
JD04	sgRNA2 rv	AATTGAGCTCCAAATAATGATTTTATTTT
JD08	OLE1::RFP fw	AATTGTTTAAACAGTGTATGTAGGTATAGTAA
JD09	OLE1::RFP rv	AATTGTTTAAACTCTAGTAACATAGATGACAC
JD125	EF1a fw	CTCACATTTTCGTAGCCGCAAGAC
JD126	EF1a rv	GATCAAGTGACCAGTTGTGGTCGAT
JD151	GABI o8409	ATATTGACCATCATACTCATTGC
JD157	CR-ISU1 fw	GAGACGAGTTATATAGTGGGAC
JD158	CR-ISU1 rv	GGATCTAAGACTAAGATGACAA
JD160	CR-ISU1 inrv	GCTGTCACACACTCTACGCCCAT
JD271	CNI1 fw	CAGTTTCCACAGTTAATCATTTGAGCGTCA
JD272	CNI1 rv	GGACGAGACACGTAGAGTTATACCAATGTG
JD335	At4g22220 BP fw	GGGGACAAGTTTGTACAAAAAAGCAGGCTCTGAATACACCCCCGACTCTTATTG
JD336	At4g22220 BP fw	GGGGACCACTTTGTACAAGAAAGCTGGGTGAGCCTGTGTGGTTTCTCCTGCTGCTG
JD337	GABI_424D02 LP	TTGATGGTCAGAACATCCTCC
JD338	GABI_424D02 RP	ATCTAGACGAAGACATTCGCC
JD368	pEC1-Cas9 fw	aattGAATTCgaataaaagcatttgcgtttggtt
JD369	pEC1-Cas9 rv	aattTTAATTAACCTAGGgttgtcaatcaattggcaagtcat
JD460	CR-At5g04560 fw	GGAGAATCAAACTCAACAAGAGTTC
JD461	CR-At5g04560 rv	GGAGAAGTCACCTCACTCCTGC
JD575	At2g21650 fw	ACATCTTTTGACGTTTTATACCCTT
JD576	At2g21650 rv	CGTCTGAAATGAGGAGTGGCT

1B

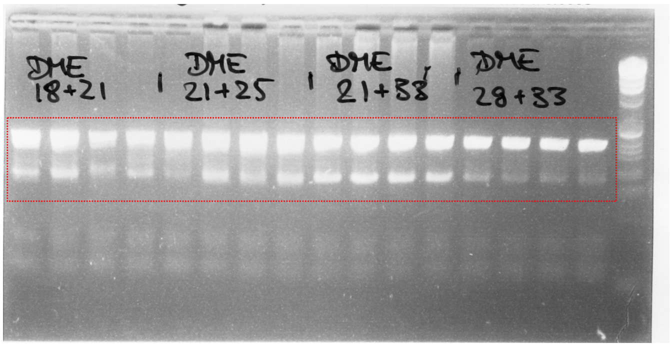

2B

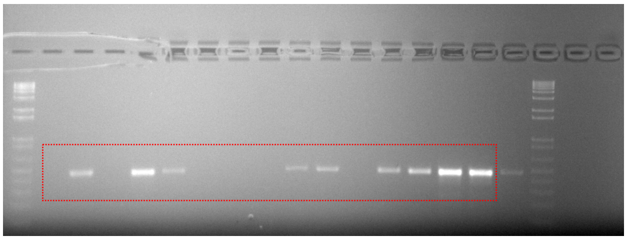

deletion

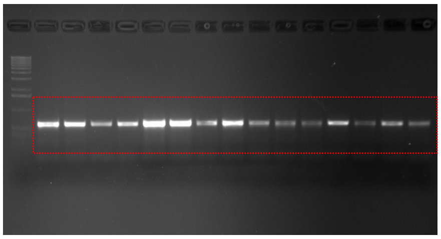

At5g60390

2F

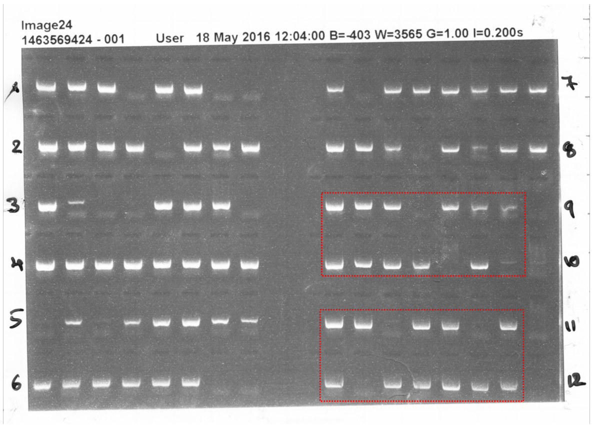

isu1-d1/*ISU1*-YFP.A8

isu1-d1/*ISU1*-YFP.E3

3B

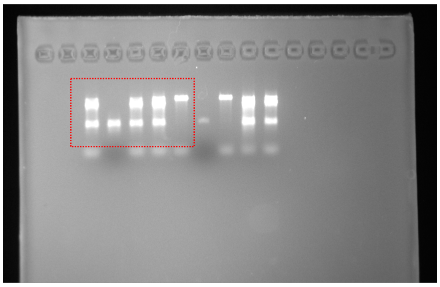

4B

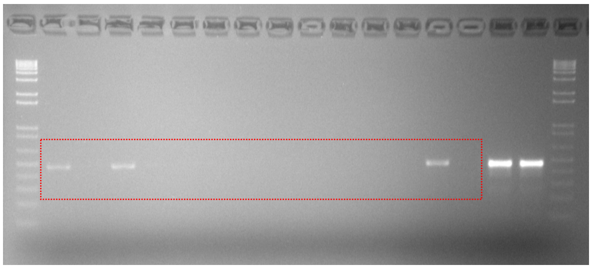

deletion

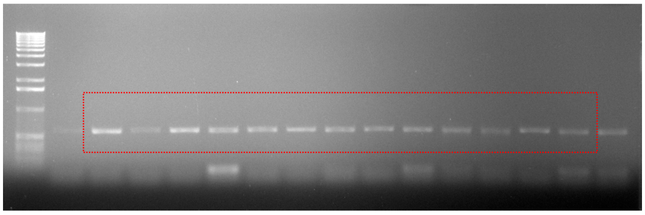

At5g60390
